# The Diatom Diversity and Ecological Status of a Tufa-Depositing River through eDNA Metabarcoding vs. a Morphological Approach—A Case Study of the Una River (Bosnia and Herzegovina)

**DOI:** 10.3390/microorganisms12081722

**Published:** 2024-08-21

**Authors:** Jasmina Kamberović, Marija Gligora Udovič, Antonija Kulaš, Kálmán Tapolczai, Sandi Orlić, Amela Jusufović, Almina Gajić, Petar Žutinić, Adisa Ahmić, Belma Kalamujić Stroil

**Affiliations:** 1 Department of Biology, Faculty of Natural Sciences and Mathematics, University of Tuzla, BA-75000 Tuzla, Bosnia and Herzegovina; jasmina.kamberovic@untz.ba (J.K.);; 2 Department of Biology, Faculty of Science, University of Zagreb, HR-10000 Zagreb, Croatia; marija.gligora.udovic@biol.pmf.hr (M.G.U.); antonija.kulas@biol.pmf.hr (A.K.); petar.zutinic@biol.pmf.hr (P.Ž.); 3 HUN-REN Balaton Limnological Research Institute, H-8237 Tihany, Hungary; 4 Institute Ruđer Bošković, HR-10000 Zagreb, Croatia; 5 Genomenon, Inc., Ann Arbor, MI 48104, USA; gajic.almina@gmail.com; 6 Society for Genetic Conservation of B&H Endemic and Autochthonous Resources, BA-71000 Sarajevo, Bosnia and Herzegovina; belma.kalamujic@ingeb.unsa.ba; 7 Institute for Genetic Engineering and Biotechnology, University of Sarajevo, BA-71000 Sarajevo, Bosnia and Herzegovina

**Keywords:** environmental DNA, Bacillariophyceae, *rbcL*, microscopy, comparative approach, karst river, diatom indices

## Abstract

Tufa deposits in karst rivers are unique habitats created by mutual interactions between specific environmental and biotope features and inhabited by diatoms as a highly abundant and diverse algal group. This pilot study aimed to investigate the diversity of diatom communities on tufa depositing habitats and assess the Una River’s ecological status using a comparative molecular and morphological approach for diatom identification. The 312 base pairs of the *rbcL* gene were barcoded and analyzed using MiSeq reads and amplicon sequence variants (ASVs) obtained by the DADA2 pipeline. The reference database Diat.barcode v7 was used for taxonomic assignment. The morphological identification of the diatoms was carried out in parallel. In total, the combined dataset revealed 46 taxa identified at genus rank, 125 on the subgenus, and 145 on combined taxonomy rank. The metabarcoding approach mostly leads to a lower number of identified taxa at species rank (58 in molecular vs. 119 in optical inventory), resulting in higher values of beta diversity and heterogeneity in diatom assemblages in samples obtained by morphological approach. Despite the high percentage of taxonomically not assigned diatom ASVs to the species rank, high Shannon diversity index values and a similar number of taxa per locations compared to the morphological approach were obtained. Taxa *Achnanthidium minutissimum* (Kützing) Czarnecki, *Achnanthidium pyrenaicum* (Hustedt) H.Kobayasi, *Amphora pediculus* (Kützing) Grunow, *Diatoma vulgaris* Bory, *Navicula cryptotenella* Lange-Bertalot, and *Navicula tripunctata* (O.F.Müller) Bory were identified at all locations in both inventories. Although limited consistency in the diatom abundances between the two inventory datasets was found, a similar grouping of samples was observed connected to the river’s longitudinal gradient. The data obtained using molecular approach in most sites indicated a mostly lower ecological status (good or moderate) compared to the data obtained from the morphological approach (high, good, and moderate). The potential of environmental DNA (eDNA) diatom metabarcoding for water monitoring and diversity studies is undeniable, but to fully realize the benefits of these methods in the future, it is essential to standardize protocols and expand the reference database for species found in specific habitats, such as tufa deposits.

## 1. Introduction

Tufa deposits and travertine occur as unique habitats in 22 European countries [1]. These habitats can be found in the karstic region in Bosnia and Herzegovina [2] and Croatia [3,4]. The process of tufa formation is determined by specific physicochemical properties (pH, temperature, calcite saturation, and calcium and magnesium concentration) and various biotope characteristics associated with the precipitation of calcium carbonate [5]. The development of ambient water travertine is influenced by aquatic mosses, cyanobacteria, and eukaryotic algae, which create biofilms that facilitate calcification. In these fragile ecosystems, diatoms are highly abundant algal taxa. Calcite precipitation by diatoms is more closely linked to their cellular products than their photosynthetic activity. In contrast, cyanobacteria exhibit calcification mostly on the extracellular sheaths surrounding their cells [6]. The variety of diatoms found in biofilms linked to tufa formation has been examined by Reichardt [7], Winsborough and Golubić [8], Plenković-Moraj et al. [9], and Žutinić et al. [10]. Further research is required to explore in more detail the diversity of diatoms inhabiting these distinctive freshwater sedimentary systems [11].

Diatoms are commonly used as a biological water quality element for evaluation of the ecological status of freshwater ecosystems [12]. The Water Framework Directive (WFD) requires biological monitoring as an integrated element in the evaluation of the ecological status of freshwaters [13]. Conventional biological monitoring and assessment techniques rely on the direct observation of organisms to compute biotic metrics/indices. However, these methods have been shown to be time-consuming and resource-intensive. Recent advancements in modern molecular methods, particularly environmental DNA (eDNA) metabarcoding combined with high-throughput sequencing (HTS), have offered a faster and more cost-effective approach to the traditional, time-consuming microscopic identification of diatoms [14,15,16,17,18,19]. Several studies have demonstrated the significant potential of eDNA diatom metabarcoding for application in water monitoring [20,21,22], but also stressed the need for standardization of methods regarding methodological uniformity of sampling, DNA extraction method, used DNA barcode, reference libraries, and bioinformatic pipelines [23,24,25,26,27,28,29].

The identification of diatoms in tufa biofilms in previous studies was mostly carried out using a morphological approach. Several recent studies have revealed the potential of applying a molecular approach to the identification of diatoms in these specific habitats [11,30], emphasizing the need for more detailed research.

The Una River is a karst river in northwestern Bosnia and Herzegovina, characterized by tufa barriers and numerous waterfalls. The specificity of the Una River is the presence of tufa-depositing formations of different ages and types: caves of tufa, travertine islands, barriers, and waterfalls [31]. A high diversity of cyanobacterial and algal taxa has been reported for the Una River in previous studies [32,33,34,35,36]. The upper part of the Una River also harbors high aquatic macroinvertebrate diversity [37] with few rare amphipods [38,39]. However, the approach of molecular identification of diatoms using eDNA metabarcoding has not yet been applied in the Una River, nor in Bosnia and Herzegovina.

This study’s objective was to explore the diversity of the diatom communities in tufa-depositing habitats, along the longitudinal profile of the Una River through a combination of molecular and morphological approaches. Additionally, we aimed to estimate the ecological status of the river based on diatom communities obtained by both approaches, using nationally proposed diatom indices in comparison with calibrated regional methodology.

## 2. Materials and Methods

### 2.1. Study Area

The Una River is a karst river with a catchment area of 9640 km^2^ that flows along a length of 212 km through the ecoregion 5 sensu Illies [40] of the Dinaric Western Balkan, mainly through the northwestern part of Bosnia and Herzegovina, with part of its course being as a border river with Croatia [41]. It springs from a strong karst spring near Donja Suvaja (375.85 m.a.s.l.) on the eastern slopes of Čemernica in Croatia and flows into the Sava River (95 m.a.s.l.). It belongs to the Black Sea basin. In the upper course, it drains a zone of high karst, while in the middle and lower part it, flows through Mesozoic limestone, dolomite, partly ophiolite, and flysch zones [42]. In the upper and middle parts, Una is a cascade river mostly flowing through the protected area of the Una National Park. The Una River’s properties are typical of tufa-depositing barriers, with a “travertine” flow of 70 km from its spring to the mouth of the tributary Sana River. The combination of the river’s different physical and chemical properties in karst ecosystems, accompanied by biological activities, causes the precipitation of carbonate minerals—calcite—and the formation of tufa deposits. The most significant amounts of travertine are present at the tectonically caused waterfalls Martin Brod and Štrbački buk in the upper reaches, with a total waterfall of 54.8 m and 23.5 m, respectively, which is why the Martin Brod area has been proposed for UNESCO’s tentative list of world natural heritage [43]. In 2008, the upper reaches of the Una River and the surrounding area were granted the status of a National Park in Bosnia and Herzegovina [44].

**Figure 1:**
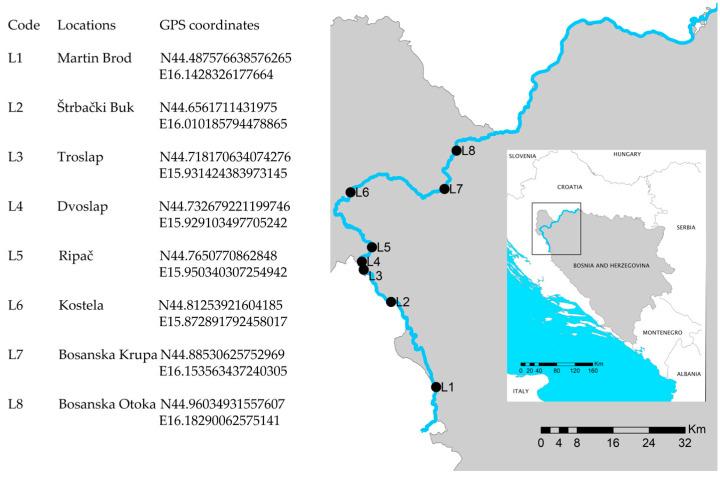
Map of sampling locations on the Una River (Bosnia and Herzegovina).

Eight locations with travertine barriers and waterfalls (L1—Martin Brod; L2—Štrbački Buk; L3—Troslap; L4—Dvoslap; L5—Ripač; L6—Kostela; L7—Bosanka Krupa; L8—Bosanska Otoka) were chosen to represent heterogeneous tufa-depositing formations across the river length profile (Figure 1). River typology was determined according to the methodology in Bosnia and Herzegovina [45] and methodology in Croatia [46].

### 2.2. Sampling

Sampling of diatoms was carried out in July 2019 during the low water period. Diatom samples were collected by scrubbing at least five collected tufa pebbles on waterfalls using DNA-free toothbrushes. Since the pebbles are often covered with moss, mosses on tufa pebbles were collected and squeezed in concentrated ethanol in a clean plastic container. Samples were preserved in a 70% final ethanol concentration and kept in 50 mL plastic vials in a fridge at +4 °C during transportation. Simultaneously, in situ measurements of physical and chemical parameters (water temperature, pH, conductivity, oxygen concentration, and saturation) were conducted using a portable multimeter 3410 (WTW Company, Weilheim, Germany). Samples for water chemistry analysis were collected and transported simultaneously with biological samples. The following parameters were quantified according to standardized methodology [47]: nitrites (NO_2_^−^-N), nitrates (NO_3_^−^-N), ammonium (NH_4_^+^-N), phosphates (PO_4_^3−^-P), total nitrogen (TN), total phosphorus (TP), silicon dioxide (SiO_2_), total organic carbon (TOC), biochemical oxygen demand (BOD), chemical oxygen demand (COD), alkalinity, turbidity (NTU), bicarbonates (HCO_3_^−^), calcium (Ca^2+^), and magnesium (Mg^2+^).

### 2.3. Microscopical and Molecular Analysis

For the purpose of morphological identification via light microscopy, diatoms were cleaned in the sulfuric acid [48] and mounted in Naphrax (Brunel Microscopes Ltd., Chippenham, UK). At least 400 diatom valves were identified in each slide using random transects under a LEICA DM 2000 light microscope (Leica Microsystems, Wetzlar, Germany) at a 1000× magnification. Identification was performed following Krammer and Lange-Bertalot [49,50,51,52], Krammer [53,54], Hofmann et al. [55], and Lange-Bertalot et al. [56]. For identification of diatoms via scanning electron microscope (SEM) type JSM-7800F (Jeol Ltd., Akishima, Tokyo, Japan), the material was filtered (Whatman filters, 3 μm pore size; Merck KGaA, Darmstadt, Germany; Whatman International Ltd., Kent, UK) and air-dried. The samples were attached by carbon tape and sputter-coated with an about 15 nm layer of platinum using the Precision Etching and Coating System, PECS II (Gatan Inc., Pleasanton, CA, USA). SEM images were recorded at a working distance (WD) of 10 mm with an electron beam voltage of 5 kV.

For the purposes of molecular identification of diatoms, DNA extraction was preceded by centrifugation (30 min at 10,000× *g*) and removal of excess water from the samples to obtain a biofilm pellet. Then, the manufacturer’s instructions were followed for the DNeasy PowerSoil Kit (Qiagen, Hilden, Germany), with modification in the last step, where DNA-Free PCR-Grade Water was added instead of Qiagen’s C6 Solution, left for 10 min and finally centrifuged. The quantity of the extracted DNA was assessed with NanoDrop spectrophotometer N660 (BioSpec—nano, Shimadzu Corporation, Kyoto, Japan).

A short part (312 base pairs) of the ribulose-1, 5-bisphosphate carboxylase large subunit (*rbcL*) chloroplastic gene was targeted as a barcode in the PCR amplification. The equimolar mix of three forward primers (Diat_*rbcL*_708F_1, Diat_*rbcL*_708F_2, and Diat_*rbcL*_708F_3) and two reverse primers (R3_1 and R3_2) was used for amplification, according to the protocol in Vasselon et al. [22]. Amplification and sequencing on the Illumina MiSeq platform were performed in the Bordeaux Transcriptome Genome Platform (PGTB, Bordeaux, France).

### 2.4. Bioinformatic Processing

The DADA2 software package [57], version 1.14 in the R programming environment, version 3.6.0 [58] was used to infer amplicon sequence variants (ASVs) from demultiplexed MiSeq reads (one R1 and one R2 fastq file per sample). The methods described in Callahan et al. [57] were applied with a slightly modified version of the original pipeline adapted to diatom metabarcoding data and available on GitHub [59]. The cutadapt command [60] was applied to remove primer sequences from R1 and R2 reads, followed by truncation of reads to 200 and 170 nucleotides, respectively, to remove the last poor-quality nucleotides. The quality profiles of the forward and reverse reads are shown in Figure S1. R1 and R2 reads with 0 ambiguities (“N”) and a maximum of expected errors (maxEE) of 2 were conserved. A parametric error model, implemented in the pipeline, was also carried out, as every amplicon dataset has a different set of error rates. The error rates for each possible transition (e.g., A→C, A→G) are shown in Figure S2. Following the dereplication of R1 and R2 reads into individual sequence units (ISUs), amplicon sequence variants (ASVs) were selected based on the error rates model determined by the DADA2 denoising algorithm. Paired reads were merged into one sequence, and chimeras were removed from the dataset. The RDP naive Bayesian classifier method [61] was used, applying the k-mer profiles of the sequences to be classified and compared against the profiles of all the sequences in a training set of sequences with assigned taxonomies. The reference sequence with the most similar profile was used to assign taxonomy to the query sequence, and a bootstrapping approach with a minimum bootstrap value of 75 was used to assess the confidence assignment at each taxonomic level. Species assignment of ASVs was performed using exact matching against the Diat.barcode database (version 7) [62,63]. For further analysis, we considered only taxa assigned at the species or at the genus level when species level was not possible to assign. Raw data in the form of the demultiplexed reads were deposited at the ENA’s Sequence Read Archive under project number PRJEB75497.

### 2.5. Statistical Analysis

The maps shown were created using ArcGIS Online software [64]. Statistics were performed using statistical software PRIMER v7 for Windows (Primer E Ltd., Auckland, New Zealand) [65], Past software version 4.16 [66], and IBM SPSS Statistics for Windows version 22.0 (IBM Corp., Armonk, NY, USA). Venn diagrams were used to illustrate the overlap in genus and species ranks identified in both inventories. The molecular data for statistical analyses were normalized through the center log-ratio transformation [67]. Morphological data (valve counts and relative abundances) were not transformed. Alpha, beta, and gamma diversity were calculated following Rimet et al. [29]. In this approach, alpha and gamma diversity are represented by the number of identified taxa (S) and the Shannon index (*H*’) is calculated using natural logarithms. Beta diversity for each location was determined by taking the ratio of the river’s gamma diversity and the alpha diversity of the specific site. Student’s *t*-test was applied to test the differences in diversity values of optical and molecular data inventories. Non-metric multidimensional scaling (NMDS) and hierarchical group average clustering using the Bray–Curtis (BC) index were utilized to examine changes in community composition associated with locations along the downstream flow direction for both the optical (OI) and molecular (MI) datasets. Additionally, the Pearson correlation coefficient was used to clarify the relationship between diatom samples and environmental factors. The correlation of the relative abundance of valve counts versus the relative abundance of center log-ratio-transformed reads was tested using a Mantel test [68] with 9999 permutations.

Given that the Una River is a border river, the ecological status was assessed using a dual methodology: the intercalibrated one used in Croatia and the proposed non-intercalibrated one applied in Bosnia and Herzegovina. Considering that the *rbcL* gene copy number depends on the biovolume of the diatom cell, a correction factor (CF) [69] was applied to the abundance data (relative read number) of the molecular dataset. This adjustment was made to address quantification bias and to ensure that the molecular dataset is comparable to the morphological dataset in assessing ecological status. The CFs were extracted from the Diat.barcode database [62]. Ecological status and the pertaining Croatian intercalibrated EQR_HR_ for molecular and morphological data were assessed by calculating the TID_HR_—Croatian Trophic Diatom Index [46,70], a diatom metric modified from Rott’s trophic index [71]. Additionally, the ecological status proposed in Bosnia and Herzegovina—the mean EQR_B&H_ value, based on three indices (SI—Saprobic Index by Pantle Buck [72]; TDI—Trophic Diatom Index by Kelly et al. [73]; IPS—Pollution Sensitivity Index by Cemagref [74])—was determined according to the methodology in Federation of Bosnia and Herzegovina [75]. Indices were calculated in Omnidia 6.0.9 software [76], except for the saprobic index, which was calculated in Excel using the list of indicators by Wegl [77]. The EQR scores obtained from morphological and molecular methods were compared using Student’s paired *t*-test [78]. Additionally, the differences in EQR classes between the two identification techniques were assessed using a pairwise Wilcoxon test [79]. SIMPER analysis [80] was carried out in order to test which of the taxa most contribute to the deviation from the expected value of the morphological intercalibrated method: “positive deviation”, “negative deviation”, or “no deviation” [81].

## 3. Results

### 3.1. Physical and Chemical Properties

The physical and chemical parameters of the Una River are listed in Table 1. The values of temperature, ammonium, and nitrites increased from the first location in the downstream direction. Total organic carbon showed decrease in the downstream direction. All measured parameters indicated high physical and chemical status of the water according to the applied federal assessment methodology in Bosnia and Herzegovina. The only exception was biological oxygen consumption at locations 2 and 3, which indicated moderate and good status, respectively.

### 3.2. Morphological Identification

**Figure 2:**
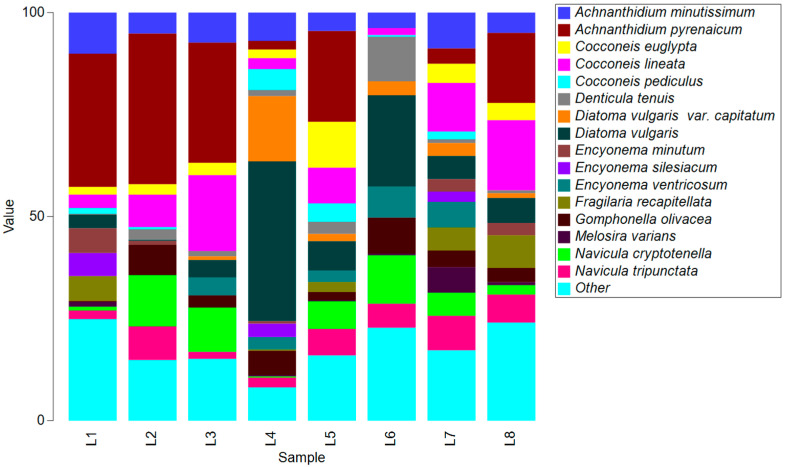
The most abundant species (more than 5%) per locations obtained by morphological approach (L1—Martin Brod; L2—Štrbački Buk; L3—Troslap; L4—Dvoslap; L5—Ripač; L6—Kostela; L7—Bosanka Krupa; L8—Bosanska Otoka).

A total of 119 diatom taxa (115 identified species) and 39 genera at eight locations were identified in the optical inventory (OI). The most abundant species overall were *Achnanthidium pyrenaicum*, *Achnanthidium minutissimum*, *Diatoma vulgaris*, *Cocconeis lineata*, and *Navicula cryptotenella*. Locations of the upper stream were specific for the higher abundance of *A*. *pyrenaicum.* The middle stream, especially location L4, is characterized by a high abundance of *D. vulgaris*, while L6 by *Denticula tenuis*. The last locations were typical for slightly more abundant *Fragilaria recapitellata*. A few taxa were constant in all samples: *A. minutissimum*, *C. lineata*, *Gomphonella olivacea*, *Navicula tripunctata*, and *N. cryptotenella* (Figure 2).

### 3.3. Molecular Approach

Sequence read numbers varied from 34,402 (L7) to 54,131 (L3) per location. A total of 333,319 reads were obtained within 265 ASVs with 255 ASVs taxonomically assigned to diatoms (Bacillariophyceae) (Table 2). Within the eight locations, a total of 32 genera (183ASVs) and 58 (133ASVs) species were identified and taxonomically classified as diatom ASVs. Of 255 diatom ASVs, 122 ASVs (47.84%) could not be classified at the species level using the Diat.barcode v7 reference database. Additionally, 72 ASVs (28.23%) were not assignable to the genus level, and 64 ASVs (25.09%) could not be classified at the family level, remaining unclassified.

**Figure 3:**
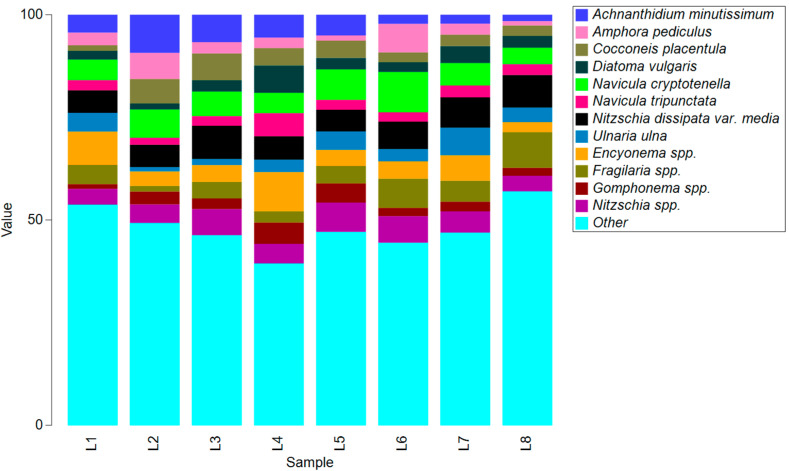
The most abundant ASVs (more than 5%) taxonomically assigned to the genera and subgenus rank obtained by molecular approach (L1—Martin Brod; L2—Štrbački Buk; L3—Troslap; L4—Dvoslap; L5—Ripač; L6—Kostela; L7—Bosanka Krupa; L8—Bosanska Otoka).

Based on the molecular approach and relative abundance of ASVs present after log-ratio transformation of read numbers, the most abundant species overall were *Achnanthidium minutissimum*, *Navicula cryptotenella*, and *Nitzschia dissipata* var. *media*, and genera *Encyonema* and *Fragilaria*. *A*. *minutissimum* were more prevalent in the upper course of the river, *Fragilaria* spp. in the middle and lower course, and *Gomphonema* spp. in the middle course. Taxa *Navicula tripunctata*, *Ellerbeckia* sp., *Gomphonema tergestinum*, and *Nitzschia fonticola* had a relatively uniform abundance longitudinally along the river (Figure 3).

### 3.4. Comparison of Two Approaches

**Figure 4:**
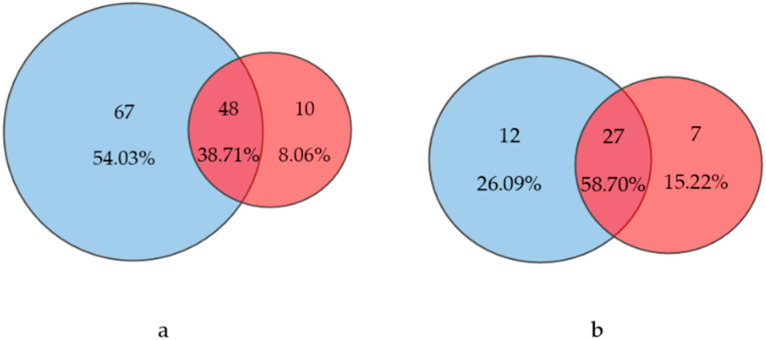
Venn diagrams showing the overlap between the number of diatoms categorized at the species level (**a**) and genus level (**b**), using either the morphological method (blue circles) or the molecular approach (red circles).

A comparison of the two approaches revealed a complete overlap of 27 genera (58.7%) and 48 species (38.71%). In general, the morphological approach discovered more taxa at genera (39) and species rank (115) than the molecular approach (34 genera and 58 species). The overlap between both approaches is illustrated by a Venn diagram (Figure 4).

**Figure 5:**
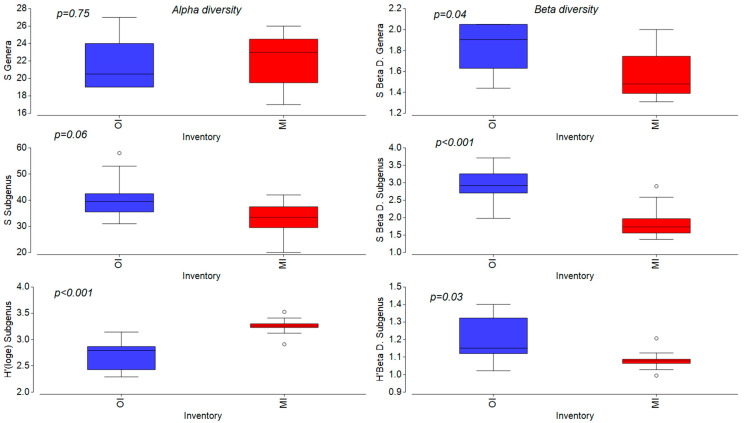
Comparison of alpha and beta diatom diversity on different taxonomic levels between OI (optical inventory data in blue) and MI (molecular inventory data in red) with shown *p*-values based on Student’s *t*-test.

In total, the combined dataset revealed 46 taxa identified at genus rank, 125 on the subgenus, and 145 on genus and species rank combined (Table 3). Taxa *Achnanthidium minutissimum*, *Achnanthidium pyrenaicum*, *Amphora pediculus*, *Diatoma vulgaris*, *Navicula cryptotenella*, and *Navicula tripunctata* were identified at all locations in both inventories. The highest numbers of subgenus taxa were obtained on site L8, Bosanska Otoka, and site L1, Martin Brod (combined approaches 68 and 64, respectively), and lowest on site L4, Dvoslap (combined 36) (Table S1). The average number of subgenus taxa per location was higher for data in the OI (S = 40.5) in comparison with data in the MI (S = 32.5). Also, more taxa identified via light microscopy were missing (10) using the molecular approach than vice versa (67). The highest difference for the subgenus level was found on sampling site 1, Martin Brod, where 36 species were not confirmed in the MI. On the genus level, the number of identified genera in the MI is similar by sampling location compared to the OI data. Genera *Aneumastus*, *Fallacia*, *Gomphonella*, *Gyrosigma*, *Humidophila*, *Meridion*, *Odontidium*, *Placoneis*, *Planothidium*, *Psammothidium*, *Pseudostaurosira*, and *Reimeria* were not assigned in the molecular analysis, while *Discostella*, *Ellerbeckia*, *Iconella*, *Lindavia*, *Mayamaea*, and *Staurosira* were not listed in the optical inventory. On the contrary, Student’s *t*-test showed significant differences (*p* < 0.01) in the two compared sets of data for Shannon diversity index, with higher values for data in the MI (avg. *H’*(log_e_) = 3.25) than in the OI (avg. *H’*(log_e_) = 2.70). Beta diversity had higher and significantly different results (*p* < 0.05) for number of genera, number of taxa on subgenus rank, and Shannon index for data obtained by morphological approach than data of the MI, thus implying greater heterogeneity in the OI dataset (Figure 5).

**Figure 6:**
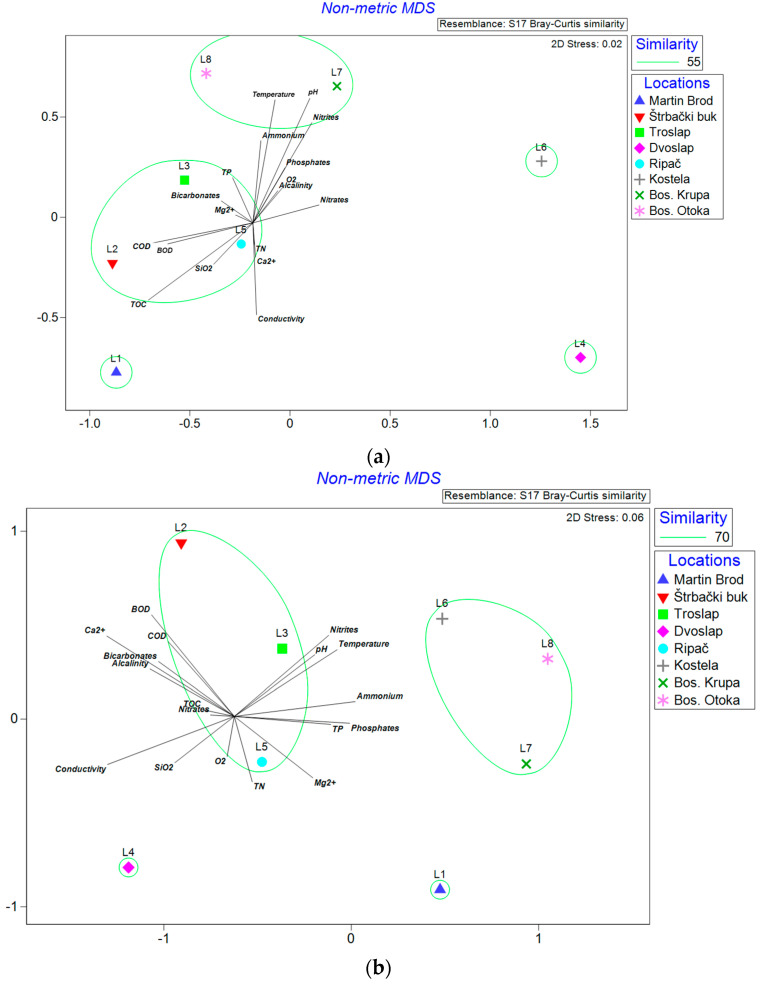
Non-metric multidimensional scaling (NMDS) of locations (location codes correspond to those in Figure 1) based on Bray–Curtis matrix of diatom assemblage similarities in relation to the applied method ((**a**)—optical inventory; (**b**)—molecular inventory), overlayed with group average clustering on presented level of similarity and Pearson correlation vectors for environmental variables (abbreviations: TN—total nitrogen; TP—total phosphorus; TOC—total organic carbon; COD—chemical oxygen demand; BOD—biological oxygen demand; SiO_2_—silicon dioxide, Ca^2+^—Calcium ion, Mg^2+^—Magnesium ion, O_2_—Oxygen concentration).

Non-metric multidimensional scaling (NMDS) of the locations, based on Bray–Curtis’s similarity distance of matrix of the taxonomic composition of diatoms, showed that the stress value was slightly lower (2D stress 0.02) for data in the OI than for data in the MI (2D stress 0.06) (Figure 6a,b). The Mantel test revealed a non-significant negative correlation for 48 species comparisons between Bray–Curtis distance derived from the relative abundance of valve counts and the relative abundance of reads (*r* = −0.1225, *p* = 0.9516). By fitting the environmental variables into the NMDS analysis and 55% similarity based on the group average clustering, samples in the OI were separated into two groups mainly along the longitudinal profile, and three individual samples (L1, L6, and L4). Samples from the MI were separated similarly in two groups and two individual samples (L4 and L6), but at a higher level of similarity (70%) than data from the OI. Higher heterogeneity in diatom assemblages in samples in the OI in comparison with the MI was detected. However, the method maintained a similar grouping of the samples, mainly following the longitudinal gradient. The comprehensive strength of the correlations between the optical and molecular inventories of the diatom community and its physical and chemical parameters is summarized in Table S2. The main parameters that showed significant (*p* < 0.01) correlation between plots based on data from the OI and the MDS1 axis were COD (*r =* −0.64), BOD (*r =* −0.56), and TOC (*r =* −0.69), and between the OI and MDS2 axis were temperature (*r =* 0.82), pH (*r* = 0.83), ammonium nitrogen (*r =* 0.54), ammonium (*r =* 0.54), nitrite nitrogen (*r =* 0.66), and nitrites (*r =* 0.66). The NMDS analysis based on the MI indicated that parameters temperature (*r =* −0.61), conductivity (*r =* 0.76), ammonium nitrogen (*r =* 0.72), ammonium (*r =* 0.72), nitrite nitrogen (*r* = 0.56), nitrites (*r =* 0.56), phosphates (*r =* −0.69), and Ca^2+^ (*r =* 0.76) had significant correlation with the MDS1 axis (*p* < 0.01), and no parameter had a significant correlation with the MDS2 axis. Results of the correlation between physical and chemical parameters in NMDS are presented in Supplementary Table S2.

The first location had the highest number of taxa sampled on the most active tufa deposits in the Una River, which typically have high abundances of *A*. *pyrenaicum*, *A*. *minutissimum*, and *Encyonema* spp., and the fourth location had the lowest number of taxa, with a typical predominance of *Diatoma vulgaris*, *Encyonema* spp., and *Gomphonella olivacea*; they were separated individually in both inventories. The group of midstream samples in the OI showed typical higher abundances of *Achnanthidium pyrenaicum*, *Cocconeis lineata*, and *Navicula cryptotenella* (the cumulative contribution to samples 2, 3, and 5 obtained by Simper analysis was up to 65 %). The MI revealed the following taxa with contributions of up to 30% for this group: *Navicula cryptotenella*, *A. minutissimum*, *Nitzschia dissipata* var. *media*, and *Nitzschia* spp. Midstream locations correlated with conductivity, COD, and BOD. Downstream locations correlated with temperature, pH, and nitrites. The optical inventory of downstream locations had the typical higher abundances of *Cocconeis lineata*, *Fragilaria recapitellata*, *Navicula tripunctatai*, and *Diatoma vulgaris* (the cumulative contribution to samples 7 and 8 obtained by Simper analysis was up to 50%). In the MI, taxa *Nitzschia dissipata* var. *media*, *Fragilaria* spp., *Navicula cryptotenella*, and *Nitzschia* spp. cumulatively contributed up to 30% to the group of downstream samples.

### 3.5. Comparison of Methods for Evaluation of Ecological Status

Ecological analysis of diatom community according to Van Dam et al. (1994) indicated dominance of alkaliphilic, oligohalobous, oxybiontic, ß-mesosaprobe, and mesotrophic taxa in the Una River. Based on the intercalibrated Croatian Trophic Diatom Index (TID_HR_), the ecological status of the Una River was classified as high (locations 1, 3, and 5) and good (remaining locations) based on data in the OI. Applying the TDI_HR_ to the data in the MI, the ecological status was good along the longitudinal profile. The methodology used in the Federation of Bosnia and Herzegovina (EQR_B&H_) is based on the calculation of the mean EQR value for three indices (SI—Saprobic Index by Pantle Buck, 1955; TDI—Trophic Diatom Index by Kelly et al. (2001); IPS—Pollution Sensitivity Index by Cemagref, 1982) and is still not intercalibrated. The EQR_B&H_ calculated on data of the OI indicated good status, except sites L4, L6, and L7, for which it was moderate, whilst the EQR_B&H_ calculated on the data of the MI indicated moderate status along the longitudinal profile (Table 4). In general, the MI in most locations indicated a mostly lower status than the OI. The worst water quality in both inventories was indicated by EQR on the basis of the TDI.

Both EQR_HR_ and EQR_B&H_ scores calculated from the taxa lists of the OI and MI showed significant differences (Student’s paired *t*-test, *p* < 0.01) and low correlation between the two applied approaches (Pearson correlation: *r* = 0.426, *p* > 0.05; *r* = −0.121, *p* > 0.05, respectively). A pairwise Wilcoxon test indicated that the two inventories generated no significantly different ecological status for the applied EQR_HR_ (*p* > 0.05), and significantly different ecological status classes according to the applied EQR_B&H_ methodology (*p* < 0.05). The Pearson correlation indicated that the following parameters significantly and negatively correlated with the observed EQR scores obtained from the MI: nitrites and EQR_IPS_ (*r* = −0.744, *p* = 0.034), COD and EQR_SI_ (*r* = −0.713, *p* = 0.047), and bicarbonates and EQR_SI_ (*r* = −0.800, *p* = 0.017). Positive and significant correlation was established for OI data between TOC and EQR_TDI_ (*r* = 0.827, *p* = 0.011), TOC and EQR_IPS_ (*r* = 0.839, *p* = 0.009), and consequently TOC and EQR_B&H_ (*r* = 0.845, *p* = 0.008).

In the comparison of the two applied methodologies for the assessment of ecological status (EQR_HR_ and EQR_B&H_), a significant positive correlation was established only for the data of the OI (*r* = 0.860; *p* = 0.006). Given that the EQR_HR_, based on a morphological approach, is an intercalibrated ecological quality ratio, the deviation of EQR_HR_ values for the molecular dataset was analyzed further. A negative deviation in the interpretation of the ecological status for one class lower was shown for locations 1, 3, and 5, while the other samples were consistent with the valorization of the status based on the morphological identification of taxa. The Simper analysis highlighted the species that are most likely contribute to a good EQR status via morphological assessment of cumulative contribution in samples up to 75% as follows: *Achnanthidium pyrenaicum* (44.03%), *Achnanthidium minutissimum* (9.74%), *Cocconeis lineata* (9.12%), *Diatoma vulgaris* (6.49%), and *Navicula cryptotenella* (5.14%).

## 4. Discussion

### 4.1. The Diatom Communities of Tufa Deposits

Tufa formations are unique habitats that result from the deposition of dissolved calcium carbonate in water, facilitated by plants, algae, and mosses. Besides cyanobacteria, diatoms are the most dominant primary producer in biofilms on tufa deposits [11,82,83]. Diatoms contribute to the maintenance of tufa-forming habitats, and calcite precipitation associated with their cell products has been reported in previous studies on tufa deposits for several genera (*Amphora*, *Gomphonema*, and *Nitzschia*) and species (*Achnanthidium affine*, *Achnanthidium minutissimum*, *Achnanthidium pyrenaicum*, *Fragilaria vaucheriae*, *Cymbella affinis*, *Encyonopsis microcephala*, and *Gomphonella calcarean*) [6], most of which were identified in this study.

The diatoms of the Una River, the largest karst river in Bosnia and Herzegovina, have so far only been studied using a morphological approach. The work by Redžić [36] lists a total of 128 diatom taxa, while the study by Hafner [34] lists 126 diatom taxa, mainly oligosaprobic and betamesosaprobic species, which is less than the 145 taxa identified in this study by a combined OI and MI. The largest differences in species composition compared to the 1990s period are observed in the previously more abundant species from the genera *Cyclotella*, *Campylodiscus*, *Diatoma*, *Diploneis*, *Epithemia*, and *Surirella*, compared to the current results. Some of these differences are caused by changes in the taxonomy of species, but also by changes in the composition of communities due to the effect of environmental factors. The morphological approach in this study shows a different species composition, especially in the genera *Achnanthidium*, *Cocconeis*, *Amphora*, and *Caloneis*, which could be a consequence of later differentiated species in these genera compared to the period of previous studies. For example, the species *Achnanthidium pyrenaicum* is predominant in these waterfalls, and in earlier morphological studies, it was probably assigned to the linear–lanceolate species of the genus *Achnanthes*. The species are adapted to fast-flowing water and are often found in rheocrenic karst springs [84]. The middle stream of the Una River was dominated by *Navicula cryptotenella*, *Diatoma vulgaris* and *D. vulgaris* var. *capitatum*. The high abundance of *N. cryptotenella* is consistent with similar studies on tufa waterfalls, as it was also found on tufa-forming biofilms in German karstic rivers [11].

In comparing diatom communities in the Una River with those in the nearby karstic river Krka [30], also with typical travertine barriers, a greater number of taxa were found in Krka River, which was investigated in several different seasons. The two rivers share common species *Achnanthidium minutissimum*, *A. pyrenaicum*, *Cocconeis lineata*, *C. euglypta*, *Encyonema minutum*, *Fragilaria gracilis*, *Gomphonema lateripunctatum*, *Meridion circulare*, *Navicula tripuncatata*, and *Rhoicosphenia abbreviata*. However, several very abundant taxa in the Krka River were not found in the Una River: *Achnanthidium aff. affine* (Grunow) Czarnecki, *Aulacoseira granulata* var. *angustissima* (O. Müller) Simonsen, *Denticula kuetzingii* Grunow, *Fragilaria paludosa* (Meister) Lange-Bertalot and S. Ulrich, *Planothidium hauckianum* (Grunow) Bukhtiyarova, and *Staurosirella pinnata* (Ehrenberg) D.M. Williams and Round.

### 4.2. Differences in Methods

In comparing two different methods of identification of diatoms, a weak overlap was found in the composition and relative abundance of common taxa from the OI and MI. Similar conclusions were given by Kulaš et al. [30] and Nistal-García et al. [85], mainly explaining the discrepancies by the incompleteness of the reference database for all identified species in the OI. This reason can also be observed in species that were very abundant in the slides but are not present in the reference database, such as *Cocconeis lineata*, *Fragilaria recapitellata*, and *Gomphonella olivacea*. Taxa that until recently were described under varieties or are still considered varieties were also not detected by molecular analysis due to the missing barcodes in the database under the correct taxonomy, although they were highly represented in the OI, such as *Cocconeis euglypta* and *Diatoma vulgaris* var*. capitatum*. The application of the molecular approach and reference database in identification of diatoms on tufa deposits in this study resulted in a weaker taxonomic resolution than for the morphological approach, especially in calcium-preferring genera *Diploneis*, *Cymbella*, and *Caloneis*; the cryptic genus *Cocconeis*; and the genera *Gomphonema*, *Fragilaria*, *Encyonema* and *Navicula*, where a large number of ASVs remained unassigned at the species level. On the other hand, the incompleteness of the reference database is not the only reason for the discrepancies, as the impossibility of detecting certain species was observed despite their presence in the reference database, e.g., *Achnantidium straubianum*, *Amphora copulata*, *Encyonema ventricosum*, *Meridion circulare*, and *Gyrosigma* spp. It could be that some species are represented by only one or a few barcodes in the database (e.g., *M. circulare*), which led to a poor match with the database. This may be caused by a possible different geographical variant of these taxa found in habitats other than tufa formations, which implies the need for more detailed research for the purposes of isolation, clonal culturing, and barcode storing of the abovementioned species and genera: *Fragilaria*, *Encyonema*, *Gomphonema*, *Diploneis*, *Navicula*, and *Nitzschia.* Reasons for detected discrepancies may also be related to the different steps in the methodology, from extraction and PCR amplification to bioinformatic processing in the molecular identification of species. The extraction of DNA from diatoms is demanding due to the different thicknesses of silicate cell walls [86], and PCR reaction can be easily inhibited by calcium [87], whose higher concentration is typical of karst rivers [30]. Bailet et al. [26] concluded that different bioinformatic pipelines show weak correspondence of the taxonomic assignments despite applying the same dataset and reference database, emphasizing the need for a future standardization of pipelines for data analysis.

Despite a large number of unassigned ASVs (122 ASVs, or 47.84%), the molecular approach in certain genera, e.g., *Nitzschia*, was more successful in detecting a higher number of species. In the case of morphologically demanding species for identification, the molecular approach has a great advantage, and in this study revealed the presence of the species *Nitzschia draveillensis*, *Nitzschia amphibia*, *Sellaphora lanceolata*, *Lindavia radiosa*, *Mayamaea permitis*, *Gomphonema affine*, and *Discostella nipponica*, which were not recorded in the OI here, nor in earlier studies. Taxa that are small in size, low in density, and with poorly expressed morphological parameters and fragile shells that can be destroyed in the preparation process of permanent slides (e.g., *Mayamaea permitis*) are often overlooked in the OI, and the molecular approach of identification gives better results compared to the light microscopy method [88]. The advantages of DNA metabarcoding compared to the morphological approach have also been observed in the detection of genera *Iconella*, *Ellerbeckia*, and *Staurosira*, with a high number of reads, especially for *Ellerbeckia*. A high number of reads can be related to cell size, as suggested by Mora et al. [89], emphasizing that cell size could be an important factor for a higher number of reads in the dataset. However, according to the applied methodology, these taxa were not detected by morphological analysis, proving that DNA metabarcoding very successfully complements the classical analysis of diatom identification.

### 4.3. Diversity of Diatoms

Alpha diversity expressed as the number of taxa was higher in the OI, which is consistent with the research of specific river ecosystems such as non-perennial rivers and streams in extreme hydrological conditions [90]. On the contrary, the Shannon diversity index had higher values for the data from the MI than the OI, which may be related to the equation that uses the natural logarithm for species abundance and thus takes the representation of rare species and the uniformity of abundance into consideration when determining the value of the index. The data for the molecular dataset were log-ratio-transformed, which reduced the differences in the number of reads for very numerous and poorly represented taxa, which could also have influenced the higher values of the Shannon diversity index for the MI. In general, alpha diversity values in molecular identification depend on the ability of the HTS method to detect rare and cryptic species and the quality of sequencing and bioinformatic processing [29]. Similar to cited research, higher values of beta and gamma diversity for the OI indicate greater heterogeneity in sample composition compared to the MI.

### 4.4. Communities in Different Environmental Conditions

Non-metric multidimensional scaling (NMDS) and group average clustering showed a similar grouping of samples, but at a higher level of similarity in the molecular dataset compared to the OI. This grouping mainly followed deviations in the number and composition of species at the first and fourth location and the longitudinal grouping of middle- and lower-stream samples. The first location, Martin Brod, is specific for the highest number of taxa, and is characterized by pronounced tufa formation processes. This site features an impressive 800 m stretch of waterfalls and cascades, boasting a vertical drop of 54 m. It is the largest and longest waterfall complex in the National Park, which is why it has been nominated for inclusion on UNESCO’s tentative list of World Heritage sites [43]. Compared to the others, this location is characterized by lower values of temperature, phosphates, nitrites, and slightly elevated values of TOC, followed by species adapted to fast water flow (*Achnanthidium* spp.) and genera *Encyonema*, *Fragilaria*, and *Nitzschia*. A total of 67% of ASVs at this location remained unsigned to the species level, indicating a potentially high hidden diversity of taxa at this location. The fourth location (Dvoslap) also remained ungrouped. Although none of the examined physicochemical parameters at this location deviate from the high physicochemical status of the water, identified dominant species *Diatoma vulgaris* and *Gomphonela olivacea*, according to van Dam et al. [91], refer to alkalibiontic, well-oxygenated, β-mesoprobic, and meso-eutraphentic habitats. As diatoms on tufa deposits are closely related to mosses as the dominant plant cover, they may have an additional input of nutrients due to close interaction with the mosses, especially in the early stages of colonization [92]. This may be the reason for the presence of meso-eutraphentic taxa in the water of high physical and chemical status. Although the physical and chemical parameters, except for biological oxygen consumption, indicated high-quality water status along the longitudinal profile, slightly higher temperature, nitrate, and ammonium ion values were observed in the middle and lower flow compared to the upper flow. This was accompanied by a greater participation of eutraphentic, β-mesoprobous, and alkaliphilous taxa*: Cocconeis lineata*, *Navicula cryptotenella*, *Nitzschia dissipata* var*. media*, *Fragilaria recapitellata*, *Navicula tripunctata*, and *Diatoma vulgaris*, which may imply greater anthropogenic pressure in the middle and lower reaches of the river.

### 4.5. Assessing the Ecological Status of the Sampling Sites

By comparing the application of two sets of data in the evaluation of ecological status, in the case of the calibrated TID_HR_, despite the weak correlation in the index values obtained from the two inventories, the ecological status did not show statistically significant differences. The results from the MI compared to the OI had one class lower status in approximately 30% of the locations. Similar results were obtained in the Joint Danube Survey by Tapolczai et al. [93], where the metabarcoding-based IPS covered a higher range of quality classes indicating lower values for downstream sites and the tributaries of the Danube River in comparison with microscopy-based IPS values.

The complementarity of the application of the list of species obtained by HTS with data from morphological analyses for the purpose of monitoring has so far been suggested in several papers (e.g., [92]), but results on deviations between the two interpretation methods are not rare either (e.g., [81]). Most often, the reasons for these deviations are the impossibility of identifying indicator species due to the poor filling of reference databases with specific species that, due to their high abundance, have been singled out by statistical methods as significant contributors to a certain ecological status [30]. The reason for the discrepancies can also be found in the mismatched relative abundances of the species on which the calculations of the diatom indices are based. In this study, Simper analysis singled out several species that contributed the most to the difference in ecological status assessment between the inventories. Similarly to the research of Bailet et al. [81], the species *Achnanthidium minutissimum* and *Cocconeis* spp. contributed to the underestimation of the ecological status using molecular identification, with higher abundance values of *A. minutissimum* in the MI and the absence of *C. lineata* from the reference database. Also, the species *D. vulgare*, *A. pyrenaicum*, and *N. cryptotenella* were less represented in the MI compared to the OI, which may result in differences in the calculation of diatom indices. In the case of the EQR_B&H_, a greater discrepancy and generally lower ecological status was observed for the data obtained by the molecular approach. Also, both sets of data revealed mostly lower status for the EQR_B&H_ in comparison with the calibrated EQR_HR_. The TDI—which, according to the B&H methodology, was proposed to be used along with the other two indices, IPS and SI—had the greatest deviation in relation to the physicochemical parameters and the values of other indices. This index was developed to investigate the impact of wastewater in England [73,94], and when applied to our data did not show sensitivity for the application in clean karst rivers, which is why its application in routine monitoring in Bosnia and Herzegovina should be revised. In routine monitoring, the Una River is investigated at three locations in the Federation of Bosnia and Herzegovina in the upper, middle and lower reaches. Similar to these studies, the results based on the phytobenthos point to a good water status, and the results of the physicochemical parameters to a high status, especially in the upper reaches [75]. A challenge in the application of DNA metabarcoding in monitoring is the method of calculating the abundance in species. Diatom indices mostly rely on abundances data that are highly dependent on the applied bioinformatics processing. In our study, we applied the CF factor to take into account the influence of cell volume on the number of reads. However, this is not the only cause that can result in a higher number of reads for certain species. As Bailet et al. suggested [26], diatom metabarcoding for ecological evaluation may also have a bright future if new metrics are created that employ presence/absence data rather than relative abundance. The application of taxonomic assignments of sequences ultimately relies on the morphological identification of species present in reference databases. As suggested by Kochoska et al. [95], it is challenging to link barcodes to matching morphological species when many clade-related environmental sequences and physically identical species coexist. For a more successful use of metabarcoding, the reference database should be completed with sequences originating from specific geographic areas and habitats, e.g., tufa-depositing rivers in this case. Recently, a new approach, the “taxonomy-free method”, was tested by Tapolczai et al. [28]. This method suggests an alternate strategy to prevent information contained by “unassigned” sequences from being lost in the case that molecular taxonomic units, e.g., ASVs, are not assigned to taxa. Although the method for studying periphytic communities in streams is relatively new, it has already offered intriguing insights into intraspecific variability and has the potential to be used in biomonitoring and bioassessment.

## 5. Conclusions

The combined approach of diatom identification contributes significantly to the alpha diversity of tufa habitats. On the other hand, beta diversity indicated greater heterogeneity when the morphological approach was used. The datasets showed similar clustering along the longitudinal gradient of the river with similar correlations to physical and chemical parameters. Although specific microhabitats such as active tufa formations require better fulfillment of reference diatom species databases for a more comprehensive biodiversity assessment, the molecular approach proved to be applicable in monitoring, especially as a valuable complement to the classical approach to biodiversity assessment. The methodology in the Federation of Bosnia and Herzegovina, on the other hand, requires modification to the method for calculating the ecological status, especially for karst rivers.

## Figures and Tables

**Table 1:** Physical and chemical water parameters measured at the sampling stations on the Una River and river typology in Bosnia and Herzegovina (B&H) and Croatia (HR). Location codes correspond to those in Figure 1.

Code	L1	L2	L3	L4	L5	L6	L7	L8
Name	Martin Brod	Štrbački Buk	Troslap	Dvoslap	Ripač	Kostela	Bosanska Krupa	Bosanska Otoka
River typology B&H *	6	5	5	5	5	5	5	5
River typology HR *	HR-R_12	HR-R_12	HR-R_12	HR-R_12	HR-R_12	HR-R_8	HR-R_8	HR-R_8
Physical and chemical parameters								
Temperature °C	11.4	13.3	11.7	12.3	12.7	14.1	15.0	15.9
pH	7.39	7.55	7.54	7.58	7.67	7.76	7.75	7.83
Oxygen concentration (mg L^−1^)	8.88	9.06	8.94	9.26	9.31	8.9	9.37	9.19
Conductivity (µS cm^−1^)	462	458	475	473	472	454	447	450
Ammonium nitrogen, NH_4_^+^-N (mg L^−1^)	0.024	0.025	0.009	0.019	0.019	0.028	0.037	0.038
Ammonium, NH_4_^+^ (mg L^−1^)	0.0309	0.0322	0.0116	0.0245	0.0245	0.0361	0.0477	0.0489
Nitrite nitrogen, NO_2_^−^-N (mg L^−1^)	0	0.0004	0	0.0001	0.0003	0.0014	0.0007	0.0009
Nitrites, NO_2_^−^ (mg L^−1^)	0	0.0013	0	0.0003	0.001	0.0046	0.0023	0.003
Nitrate nitrogen, NO_3_^−^-N (mg L^−1^)	0.252	0.269	0.305	0.379	0.36	0.337	0.25	0.394
Nitrates NO_3_^−^ (mg L^−1^)	1.115	1.1908	1.3501	1.6777	1.5936	1.4918	1.1067	1.7441
Total nitrogen, TN (mg L^−1^)	0.4	0.35	0.397	0.395	0.395	0.381	0.355	0.41
Chemical oxygen demand, COD (mg L^−1^)	12.5	25	18.8	6.25	12.5	6.25	12.5	6.25
Biological oxygen demand, BOD (mg L^−1^)	3.1	6.8	4.8	2.5	3.4	2.7	2.9	2.5
Total phosphorus, TP (mg L^−1^)	0.028	0.017	0.027	0.018	0.003	0.02	0.029	0.027
Phosphates, PO_4_^3−^-P (mg L^−1^)	0.009	0.005	0.007	0.006	0.0011	0.01	0.011	0.008
Total organic carbon, TOC (mg L^−1^)	1.38	1.18	0.73	0.07	0	0	0	0.11
Silicon dioxide, SiO_2_ (mg L^−1^)	1.88	1.662	2.238	1.765	1.99	1.53	1.838	1.403
Alkalinity as CaCO_3_ (mg L^−1^)	214.9	227.3	238.7	231.4	233.5	225.2	227.3	225.2
Turbidity (NTU)	17.3	11.2	15	13.6	17.3	15.6	13.8	16.6
Bicarbonates, HCO_3_^−^ (mg L^−1^)	246.4	261.1	275.7	256.9	258.6	248.9	258.6	251.3
Ca^2+^ (mg L^−1^)	80.2	97.5	91.2	92.8	83.4	84.9	81.8	84.9
Mg^2+^ (mg L^−1^)	19.5	0	13.8	3.817	19.1	15.3	12.4	9.5

* type 6—small and medium-sized hill and mountain rivers with a dominance of large fractions in the bottom substrate; type 5—small and medium-sized lowland and mountain rivers with medium-coarse bottom substrate; type HR-R_12—medium and large upland rivers; type HR-R_8—lowland medium-sized rivers.

**Table 2:** Read number and number of ASVs per site.

	L1	L2	L3	L4	L5	L6	L7	L8	Total
Read number	42,602	44,938	54,131	43,975	37,312	40,172	34,402	35,787	333,319
Number of detected ASVs	79	160	101	47	103	100	75	86	265
Number of ASVs assigned to the Phylum Bacillariophyta	77	159	98	47	101	97	75	85	255
Number of ASVs assigned to the Genus taxonomic level	57	117	73	36	74	76	60	70	183
Number of ASVs assigned to the Species taxonomic level	41	88	54	25	50	53	44	53	133
Number of unique Genera	19	26	24	17	23	23	20	25	32
Number of unique Species	28	42	34	20	33	34	31	41	58

**Table 3:** Taxa list obtained in optical (OI) and molecular inventory (MI) presented as sum of counts in OI, sum of ASV reads in MI, number (No.) of ASV reads per assigned taxa, average % of similarity ASVs with the reference barcodes, abundance per location (1—Martin Brod; 2—Štrbački Buk; 3—Troslap; 4—Dvoslap; 5—Ripač; 6—Kostela; 7—Bosanka Krupa; 8—Bosanska Otoka) in the form of the highlighted ordinal scale based on relative abundances of counts for OI and raw reads for MI as follows: 0, >0, <1, 1 < 5, 5 < 10, 10 < 30, 30 < 60, >60.

Taxa Seen in Both Methods	Sum of Counts in OI	Sum of ASV Reads in MI	No. of ASVs	% of Similarity of ASVs	OI								MI							
					1	2	3	4	5	6	7	8	1	2	3	4	5	6	7	8
*Achnanthidium minutissimum* (Kützing) Czarnecki	171	101,216	10	93.7																
*Achnanthidium pyrenaicum* (Hustedt) H.Kobayasi	504	2779	2	95.5																
*Amphora ovalis* (Kützing) Kützing	4	4519	2	94																
*Amphora pediculus* (Kützing) Grunow	39	1343	7	97.7																
*Caloneis fontinalis* (Grunow) A.Cleve	1	84	4	87.5																
*Cocconeis pediculus* Ehrenberg	50	594	1	94																
*Cocconeis placentula* Ehrenberg	2	2076	8	96.6																
*Cyclotella distinguenda* Hustedt	2	130	2	100																
*Cymatopleura elliptica* (Brébisson) W.Smith	1	50	1	87																
*Cymbella cymbiformis* C.Agardh	1	45	2	88.5																
*Cymbella lanceolata* (C.Agardh) C.Agardh	1	133	1	89																
*Denticula tenuis* Kützing	62	1086	2	100																
*Diatoma moniliformis* (Kützing) D.M.Williams	15	2585	1	94																
*Diatoma vulgaris* Bory	271	62,998	2	93																
*Diploneis subovalis* Cleve	2	434	3	99.6																
*Encyonema leibleinii* (C.Agardh) W.J.Silva, R.Jahn, T.A.V.Ludwig, and M.Menezes	2	56	1	100																
*Encyonema minutum* (Hilse) D.G. Mann	50	22	1	94																
*Encyonema silesiacum* (Bleisch) D.G.Mann	43	256	1	78																
*Eolimna minima* (Grunow) Lange-Bertalot, nom. illeg.	2	14	1	100																
*Epithemia sorex* Kützing	1	233	1	92																
*Eunotia arcus* Ehrenberg	3	21	1	100																
*Fistulifera saprophila* (Lange-Bertalot and Bonik) Lange-Bertalot	1	206	2	99.5																
*Fragilaria gracilis* Østrup	1	482	2	100																
*Frustulia vulgaris* (Thwaites) De Toni	2	20	1	99																
*Gomphonema pumilum* (Grunow) E.Reichardt and Lange-Bertalot	8	626	4	89.2																
*Gomphonema saprophilum* (Lange-Bertalot and E.Reichardt) Abraca, R.Jahn, J.Zimmermann and Enke	1	71	2	98.5																
*Gomphonema micropus* Kützing	1	13	1	100																
*Gomphonema tergestinum* (Grunow) Fricke	11	377	1	100																
*Karayevia ploenensis* (Hustedt) Bukhtiyarova	4	7	1	100																
*Melosira varians* C.Agardh	30	24,297	1	100																
*Navicula antonii* Lange-Bertalot	6	256	2	98																
*Navicula cryptotenella* Lange-Bertalot	158	5812	12	93.7																
*Navicula tripunctata* (O.F.Müller) Bory	138	23,424	2	92.5																
*Navicula capitatoradiata* H.Germain ex Gasse	5	523	1	100																
*Navicula gregaria* Donkin	2	71	1	100																
*Nitzschia dissipata* (Kützing) Rabenhorst	32	103	1	93																
*Nitzschia fonticola* (Grunow) Grunow	3	2144	4	94.5																
*Nitzschia gracilis* Hantzsch	2	79	1	100																
*Nitzschia linearis* W.Smith	1	588	4	98																
*Nitzschia palea* (Kützing) W.Smith	1	142	2	100																
*Nitzschia pusilla* Grunow	1	72	3	90																
*Nitzschia sigmoidea* (Nitzsch) W.Smith	1	691	3	94.6																
*Nitzschia tubicola* Grunow	2	8	1	97																
*Nitzschia dissipata* var. *media* (Hantzsch) Grunow	8	7149	9	92.6																
*Rhoicosphenia abbreviata* (C.Agardh) Lange-Bertalot	1	6	1	98																
*Sellaphora bacillum* (Ehrenberg) D.G.Mann	1	30	1	92																
*Surirella librile* (Ehrenberg) Ehrenberg	4	183	3	93.6																
*Ulnaria ulna* (Nitzsch) Compère	7	4998	3	98.3																
**Taxa seen in OI but not in MI**	0	0																		
*Achnanthidium anastasiae* (Kaczmarska) Chaudev and Gololobova	9	0																		
*Achnanthidium straubianum* (Lange-Bertalot) Lange-Bertalot	2	0																		
*Amphora copulata* (Kützing) Schoeman and R.E.M. Archibald	8	0																		
*Amphora inariensis* Krammer	15	0																		
*Amphora lange-bertalotii* Levkov and Metzeltin	1	0																		
*Aneumastus stroesei* (Østrup) D.G.Mann	3	0																		
*Caloneis bacillum* (Grunow) Cleve	3	0																		
*Caloneis lancettula* (Schulz) Lange-Bertalot and Witkowski	5	0																		
*Cocconeis euglypta* Ehrenberg	103	0																		
*Cocconeis lineata* Ehrenberg	219	0																		
*Cocconeis neodiminuta* Krammer	9	0																		
*Cocconeis placentula* var. *klinoraphis* Geitler	15	0																		
*Cocconeis pseudolineata* (Geitler) Lange-Bertalot	28	0																		
*Cymatopleura apiculata* W.Smith	2	0																		
*Cymatopleura solea* (Brébisson) W.Smith	3	0																		
*Cymbella compacta* Østrup	1	0																		
*Cymbella excisiformis* Krammer	1	0																		
*Cymbella lange-bertalotii* Krammer	1	0																		
*Diatoma vulgaris* var. *capitatum* Grunow	83	0																		
*Diploneis fontium* Richardt and Lange-Bertalot	1	0																		
*Diploneis krammeri* Lange-Bertalot and E.Reichardt	3	0																		
*Diploneis marginestriata* Hustedt	3	0																		
*Encyonema ventricosum* (C.Agardh) Grunow	69	0																		
*Encyonema vulgare* Krammer	3	0																		
*Encyonema caespitosum* Kützing	1	0																		
*Encyonopsis microcephala* (Grunow) Krammer	1	0																		
*Eunotia* sp. Ehrenberg	2	0																		
*Fallacia subhamulata* (Grunow) D.G.Mann	2	0																		
*Fragilaria mesolepta* Rabenhorst	1	0																		
*Fragilaria recapitellata* Lange-Bertalot and Metzeltin	76	0																		
*Fragilaria vaucheriae* (Kützing) J.B.Petersen	16	0																		
*Gomphonella olivacea* (Hornemann) Rabenhorst	108	0																		
*Gomphonella olivaceolacua* (Lange-Bertalot and E.Reichart) R.Jahn and N.Abarca	5	0																		
*Gomphonema clavatulum* Reichardt	1	0																		
*Gomphonema elegantissimum* Reichardt and Lange-Bertalot	6	0																		
*Gomphonema lateripunctatum* E.Reichardt and Lange-Bertalot	13	0																		
*Gomphonema minutum* (C.Agardh) C.Agardh	4	0																		
*Gomphonema parvulum* (Kützing) Kützing	3	0																		
*Gomphonema pseudotenellum* Lange-Bertalot	2	0																		
*Gomphonema* sp. Ehrenberg	1	0																		
*Gomphonema vibrio* var. *vibrio* Ehrenberg	1	0																		
*Gyrosigma attenuatum* (Kützing) Rabenhorst	7	0																		
*Gyrosigma sciotoense* (W.S.Sullivant) Cleve	6	0																		
*Gyrosigma obtusatum* (Sullivant and Wormley) C.S.Boyer	1	0																		
*Humidophila contenta* (Grunow) Lowe, Kociolek, J.R.Johansen, Van de Vijver, Lange-Bertalot and Kopalová	1	0																		
*Meridion circulare* (Greville) C.Agardh	21	0																		
*Navicula cryptofallax* Lange-Bertalot and G.Hofmann	2	0																		
*Navicula digitoconvergens* Lange-Bertalot	1	0																		
*Navicula hintzii* Lange-Bertalot	1	0																		
*Navicula oppugnata* Hustedt	6	0																		
*Navicula radiosa* Kützing	6	0																		
*Navicula reinhardtii* (Grunow) Grunow	7	0																		
*Navicula upsaliensis* (Grunow) M.Peragallo	4	0																		
*Navicula viridulacalcis* Lange-Bertalot	2	0																		
*Nitzschia intermedia* Hantzsch	10	0																		
*Nitzschia oligotraphenta* (Lange-Bertalot) Lange-Bertalot	2	0																		
*Nitzschia sublinearis* Hustedt	1	0																		
*Odontidium anceps* (Ehrenberg) Ralfs	5	0																		
*Odontidium mesodon* (Kützing) Kützing	2	0																		
*Placoneis* sp. Mereschkowsky	1	0																		
*Planothidium dubium* (Grunow) Round and Bukhtiyarova	1	0																		
*Planothidium lanceolatum* (Brébisson ex Kützing) Lange-Bertalot	1	0																		
*Psammothidium grischunum* Bukhtiyarova and Round	1	0																		
*Pseudostaurosira brevistriata* (Grunow) D.M.Williams and Round	1	0																		
*Reimeria sinuata* (W.Gregory) Kociolek and Stoermer	3	0																		
*Sellaphora laevissima* (Kützing) D.G.Mann	3	0																		
*Sellaphora* sp. Mereschowsky	1	0																		
*Surirella angusta* Kützing	1	0																		
*Surirella brebissonii var. kuetzingii* Krammer and Lange-Bertalot	2	0																		
*Ulnaria acus* (Kützing) Aboal	3	0																		
*Ulnaria capitata* (Ehrenberg) Compère	1	0																		
**Taxa seen in OI but not in MI**	0	0																		
*Achnanthidium* spp. Kützing	0	1806	1	100																
*Amphora* spp. Ehrenberg ex Kützing	0	743	2	99																
*Caloneis* spp. Cleve	0	2033	4	94.75																
*Cyclotella* spp. (Kützing) Brébisson	0	8	1	89																
*Cymbella* spp. C.Agardh	0	263	2	95.5																
*Diploneis* spp. Ehrenberg ex Cleve	0	48	1	100																
*Discostella nipponica* (Skvortsov) A.Tuji and D.M.Williams	0	42	1	78																
*Ellerbeckia* sp. R.M.Crawford,	0	7381	1	100																
*Encyonema* spp. Kützing	0	31,058	3	100																
*Encyonopsis* spp. Krammer	0	60	1	96																
*Fragilaria* spp. Lyngbye	0	6864	6	99.3																
*Fragilaria radians* (Kützing) D.M.Williams and Round	0	28	1	83																
*Gomphonema* spp. Ehrenberg	0	2820	5	96.4																
*Gomphonema affine* Kützing	0	78	1	76																
*Iconella* spp. Jurilj	0	168	2	100																
*Lindavia radiosa* (Grunow) De Toni and Forti	0	219	1	94																
*Mayamaea permitis* (Hustedt) K.Bruder and Medlin	0	18	1	100																
*Navicula* spp. Bory	0	215	4	88																
*Neidium* spp. Pfitzer	0	14	1	98																
*Nitzschia* spp. Hassall	0	1603	13	97.2																
*Nitzschia amphibia* Grunow	0	7	1	99																
*Nitzschia capitellata* Hustedt	0	11	1	98																
*Nitzschia draveillensis* Coste and Ricard	0	205	1	96																
*Sellaphora lanceolata* D.G.Mann and S.Droop	0	9	1	94																
*Staurosira* spp. Ehrenberg	0	308	3	100																
*Surirella* spp. Turpin	0	263	1	99																

**Table 4:** Ecological status, expressed as ecological quality ratio in Croatia (EQR_HR_) and Bosnia and Herzegovina (EQR_B&H_), and Index values of the investigated locations (L1—Martin Brod; L2—Štrbački Buk; L3—Troslap; L4—Dvoslap; L5—Ripač; L6—Kostela; L7—Bosanka Krupa; L8—Bosanska Otoka) based on optical and molecular inventories: TID_HR_—Croatian trophic diatom index; SI—saprobic index; TDI—trophic diatom index; IPS—pollution sensitivity index; EQR_SI_, EQR_TDI_, EQR_IPS_—ecological quality ratio based on SI, TDI, and IPS. Color interpretation of ecological status: blue—high; green—good; yellow—moderate; orange—poor; red—bad.

Locations Morphological Approach	L1	L2	L3	L4	L5	L6	L7	L8
TID_HR_	2.19	2.34	2.30	2.55	2.30	2.72	2.51	2.29
**EQR_HR_**	**0.87**	**0.81**	**0.83**	**0.74**	**0.83**	**0.69**	**0.76**	**0.84**
SI	1.79	1.77	1.93	2.06	1.80	1.88	1.91	1.88
EQR_SI_	0.78	0.78	0.69	0.61	0.76	0.72	0.70	0.72
TDI	42.32	52.19	50.3	76.12	55.52	73.79	69.24	55.03
EQR_TDI_	0.68	0.37	0.41	0.00	0.29	0.00	0.00	0.30
IPS	17.8	18.0	17.1	16.3	17.0	16.6	15.9	17.1
EQR_IPS_	0.86	0.88	0.82	0.77	0.81	0.79	0.74	0.82
**EQR_B&H_**	**0.78**	**0.68**	**0.64**	**0.46**	**0.62**	**0.50**	**0.48**	**0.61**
Molecular approach	L1	L2	L3	L4	L5	L6	L7	L8
TID_HR_	2.44	2.70	2.64	2.43	2.48	2.80	2.74	2.62
**EQR_HR_**	**0.78**	**0.69**	**0.70**	**0.78**	**0.76**	**0.67**	**0.69**	**0.73**
SI	1.84	1.89	1.89	1.86	1.87	1.84	1.90	1.83
EQR_SI_	0.75	0.71	0.71	0.73	0.72	0.74	0.71	0.75
TDI	62.5	65.88	100	62.17	62.43	73.23	59.78	61.88
EQR_TDI_	0.20	0.05	0.00	0.14	0.13	0.00	0.19	0.14
IPS	14.3	14.4	15.3	15.3	14.6	13.2	15.0	14.3
EQR_IPS_	0.64	0.65	0.71	0.71	0.66	0.58	0.69	0.64
**EQR_B&H_**	**0.53**	**0.47**	**0.47**	**0.52**	**0.51**	**0.44**	**0.53**	**0.51**

## Data Availability

The original raw data presented in this study are openly available at the ENA’s Sequence Read Archive under project number PRJEB75497.

## Supplementary Materials

The following supporting information can be downloaded at: https://www.mdpi.com/article/10.3390/microorganisms12081722/s1, Figure S1. The quality profiles of the forward (a) and reverse (b) reads, Figure S2. The error rates for forward (a) and reverse (b) transition, Table S1. Alpha, beta, and gamma diversity on different taxonomic levels compared between OI (optical inventory) and MI (molecular inventory) given for eight sampling locations (L1—Martin Brod; L2—Štrbački Buk; L3—Troslap; L4—Dvoslap; L5—Ripač; L6—Kostela; L7—Bosanka Krupa; L8—Bosanska Otoka). S—the number of identified taxa; H’—Shannon index; D—diversity, Table S2. Pearson correlation coefficient of physical and chemical parameters for water and MDS axes. Values are in bold when *p* < 0.01 (O_2_—oxygen; TN—total nitrogen; TP—total phosphorus; TOC—total organic carbon; COD—chemical oxygen demand; BOD—biological oxygen demand; SiO_2_—silicon dioxide).
