# Supplementary material for: The Diatom Diversity and Ecological Status of a Tufa-Depositing River through eDNA Metabarcoding vs. a Morphological Approach—A Case Study of the Una River (Bosnia and Herzegovina)
Source: Microorganisms. 2024 Aug 21;12(8):1722. doi: 10.3390/microorganisms12081722 (PMC11357282; doi:10.3390/microorganisms12081722)
Supplement: Supplementary file 1 [file microorganisms-12-01722-s001.zip › microorganisms-3156001-supplementary.pdf]

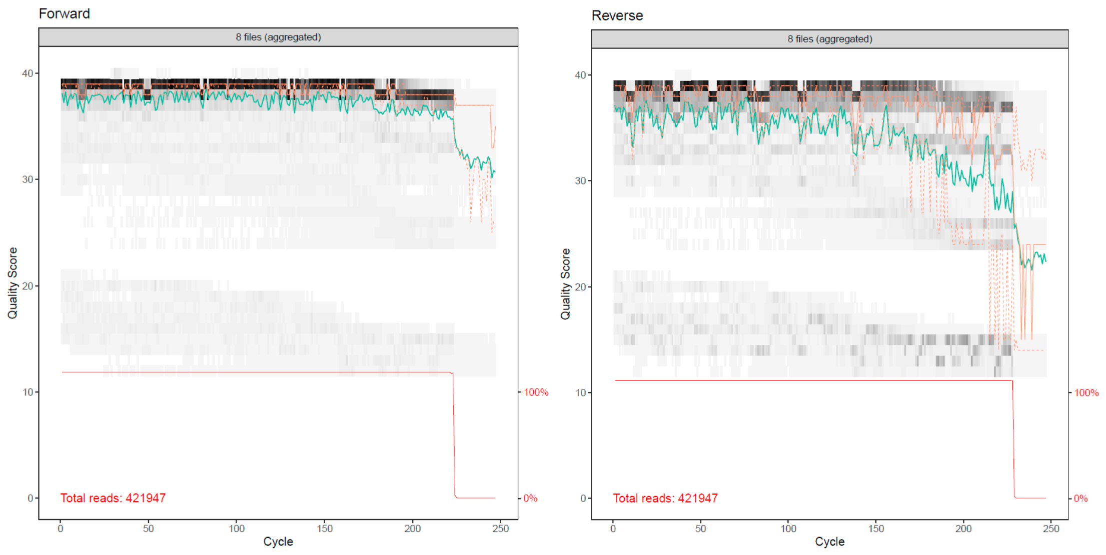

a b

Figure S1. The quality profiles of the forward (**a**) and reverse (**b**) reads

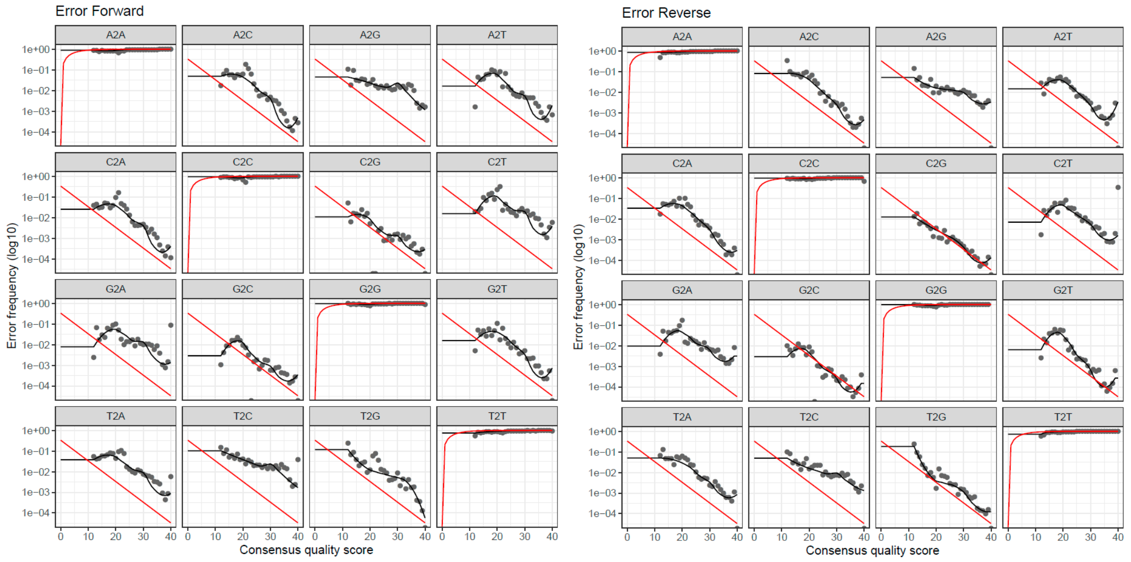

a b

Figure S2. The error rates for forward (**a**) and reverse (**b**) transition

Table S1. Alpha, beta and gamma diversity on different taxonomic levels compared between OI (optical inventory) and MI (molecular inventory) given for eight sampling locations (L1-L8), S - the number of identified taxa, *H′* - Shannon index, D. - Diversity

Locations		L1	L2	L3	L4	L5	L6	L7	L8	Arith. mean	Gamma D.
Genera	S OI	27	21	19	19	20	19	23	25	21.6	39
	S MI	19	26	24	17	23	23	20	25	22.1	34
	S Combined	30	30	30	23	30	29	27	33	29.0	46
	Missing S in OI	3	9	11	4	10	10	4	8	7.4	7
	Missing S in MI	11	4	6	6	7	6	7	8	6.9	12
	S Beta D. OI	1.44	1.86	2.05	2.05	1.95	2.05	1.7	1.56	1.83	
	S Beta D. MI	1.79	1.31	1.42	2	1.48	1.48	1.7	1.36	1.56	
Subgenus taxa	S OI	58	43	31	33	38	38	42	41	40.5	115
	S MI	28	42	34	20	33	34	31	41	32.9	58
	S Combined	64	60	54	36	62	53	60	68	57.1	125
	Missing S in OI	6	17	23	3	24	15	18	27	16.6	10
	Missing S in MI	36	18	20	16	29	19	29	27	24.3	67
	H′(loge) OI	2.84	2.40	2.45	2.31	2.84	2.76	3.14	2.89	2.70	3.21
	H′(loge) MI	3.21	3.31	3.25	2.90	3.28	3.24	3.26	3.52	3.25	3.51
	S Beta D. OI	1.98	2.67	3.71	3.48	3.03	3.03	2.74	2.80	2.84	2.92
	S Beta D. MI	2.07	1.38	1.71	2.90	1.76	1.71	1.87	1.41	1.76	1.84
	Beta D. *H′* OI	1.13	1.34	1.31	1.40	1.13	1.17	1.02	1.11	1.20	
	Beta D. *H′*(loge) OI	1.09	1.06	1.08	1.21	1.07	1.08	1.08	0.99	1.08	

Table S2. Pearson correlation coefficient of physical and chemical parameters of water and MDS axes, values are in bold when *p<0.01* (O_2 – oxygen, TN = Total nitrogen, TP - Total phosphorus, TOC = total organic carbon, COD - Chemical oxygen demand, BOD - Biological oxygen demand, SiO_2 - Silicon dioxide)

Parameters	Molecular inventory		Optical inventory	
	MDS1	MDS2	MDS1	MDS2
Temperature	**-0.619484507**	-0.39999	0.1479542	**0.820896**
pH	-0.484550523	-0.36984	0.3812047	**0.832364**
O_2	0.043193784	0.241591	0.2148577	0.25474
Conductivity	**0.762929527**	0.288928	0.0240737	-0.61302
Ammonium-nitrogen	**-0.728795312**	-0.08936	0.0520363	**0.548781**
Ammonium	**-0.72854007**	-0.08905	0.0535018	**0.548918**
Nitrite-nitrogen	**-0.562354324**	-0.48582	0.4013015	**0.663946**
Nitrites	**-0.568352538**	-0.48606	0.3945011	**0.668775**
Nitrate-nitrogen	0.144030656	-0.00789	0.442201	0.119342
Nitrates	0.144132947	-0.00831	0.4423542	0.119721
TN	-0.108388522	0.393447	0.0090393	-0.14542
COD	0.39857737	-0.44905	**-0.67425**	-0.13531
BOD	0.497923656	-0.609	**-0.568476**	-0.14037
TP	-0.581405426	0.048207	-0.135522	0.301358
Phosphates	**-0.694673793**	0.041244	0.2148408	0.368094
TOC	0.190516525	-0.04004	**-0.698619**	-0.51542
SiO_2	0.35760742	0.278355	-0.26182	-0.27579
Alcalinity	0.5073768	-0.28435	0.1665285	0.214103
Bicarbonates	0.456293438	-0.3289	-0.21079	0.14673
Ca2+	**0.766987424**	-0.48159	0.0178936	-0.22815
Mg2+	-0.472823071	0.369308	-0.116084	0.052792
